# Unraveling the Mechanisms, Clinical Impact, Comparisons, and Safety Profiles of Slow-Release Therapies in Glaucoma

**DOI:** 10.3390/pharmaceutics17050580

**Published:** 2025-04-28

**Authors:** Marco Zeppieri, Caterina Gagliano, Daniele Tognetto, Mutali Musa, Federico Bernardo Rossi, Angelo Greggio, Giuliano Gualandi, Alessandro Galan, Silvia Babighian

**Affiliations:** 1 Department of Ophthalmology, University Hospital of Udine, 33100 Udine, Italy; 2 Department of Medicine, Surgery and Health Sciences, University of Trieste, 34127 Trieste, Italy; 3 Department of Medicine and Surgery, University of Enna “Kore”, Piazza dell’Università, 94100 Enna, Italy; 4 Mediterranean Foundation “G.B. Morgagni”, 95125 Catania, Italy; 5 Department of Optometry, University of Benin, Benin 300238, Nigeria; 6 Africa Eye Laser Centre, Km 7, Benin 300105, Nigeria; 7 ARHYVAB, International PhD Program in Arterial Hypertension and Vascular Biology, Department of Medicine—DIMED, University of Padua, 35122 Padova, Italy; 8 Department of Ophthalmology, Ospedale Sant’Antonio, Azienda Ospedaliera, 35127 Padova, Italysilvia.babighian@aopd.veneto.it (S.B.)

**Keywords:** glaucoma, intraocular pressure, drug delivery systems, efficacy, patient adherence, biocompatibility

## Abstract

Glaucoma, a primary cause of irreversible blindness, is most effectively managed by reducing intraocular pressure (IOP). Topical eye drops, which are conventional treatments, frequently encounter constraints regarding patient compliance, inconsistent dosage, and tolerability. Slow-release drug delivery systems have emerged as a promising innovation in response to these challenges. The objective of these systems is to enhance the efficacy of treatment and patient compliance by ensuring the consistent and sustained delivery of therapeutic agents over extended periods. Implantable devices, injectable formulations, and external applications are all categorized as slow-release therapies. By delivering medication directly to the target tissues in a controlled manner, these technologies have the potential to circumvent common issues associated with traditional regimens, such as forgotten doses or improper administration. These systems have been shown to obtain clinically meaningful reductions in IOP in studies, with some demonstrating efficacy that is comparable to that of established daily topical treatments. Despite their potential, slow-release therapies encounter obstacles that necessitate resolution. Potential complications during implantation or removal, long-term biocompatibility, and the cost of treatment are all areas of concern. Furthermore, further investigation is required to comprehensively assess their relative economic feasibility, patient acceptability, and long-term safety profiles in comparison to conventional treatments. This review summarizes the most recent findings in the scientific literature, underlining the role and possible limits of slow-release therapies in glaucoma with the aim of offering a comprehensive understanding of their potential clinical applications and challenges. This emphasizes the potential for these innovations to revolutionize care by addressing current knowledge gaps, while also emphasizing the areas in which further development and research are required.

## 1. Introduction

Glaucoma is the second cause of blindness in the world, affecting almost 100 million people, with numbers expected to increase further in the next decade [1,2]. Furthermore, even though a wide array of effective active principles, mainly in the form of eye drops, are currently available for the treatment of glaucoma, acting through different mechanisms to control the only modifiable contributing factor to the disease, that is ocular hypertension (OHT), overall trends of disease control remain widely unsatisfactory worldwide.

Major hurdles in the clinical management of glaucoma involve patient compliance. Many therapeutic combinations require the installation of multiple eyedrops at different times of the day, and patients must make frequent follow-up visits to their eye-care provider, further straining an already burdened healthcare system. While new techniques of minimally invasive glaucoma surgery (MIGS) are also being developed with promising results, they currently remain limited to the expertise of a few selected centers worldwide. They are usually reserved for resistant, non-compliant patients [3].

Five decades ago, the first long-acting drug delivery system (LADDS) was developed and approved by the FDA for glaucoma treatment. It provides a simple solution to slow or prevent disease progression. Even though the original device was eventually discontinued by the manufacturers two decades later, technological advances in the last few years have sparked a new light on the possibility of new sustained-release devices. Thus, this review aims to update readers on the latter devices and will focus particularly on those that have recently received FDA approval for the treatment of glaucoma or are in an advanced stage of clinical experimentation, with a particular focus on advances made in the last five years.

Honorable mentions will also be made to particularly promising technologies that hold great potential for future clinical applicability as possible solutions to the remaining existing challenges.

## 2. Materials and Methods

We reviewed publications on ocular slow-release therapies on PubMed (https://pubmed.ncbi.nlm.nih.gov/, accessed on 1 March 2025) published from 2015 to date (10 years). We utilized a systematic strategy to collect and analyze pertinent papers regarding slow-release treatments for glaucoma. A literature search was performed specifically targeting articles concerning ocular slow-release treatments. The search approach incorporated keywords including “ocular”, “release”, and “therapies”, along with any pertinent terms and synonyms to guarantee a thorough search.

The preliminary search produced 108 items, which were further evaluated for relevancy according to their titles and abstracts. Subsequent to this screening, 63 records were removed for irrelevance to the review’s scope. Consequently, four records were eliminated due to linguistic limitations, while seven additional studies were omitted due to the unavailability of full texts. The rigorous screening process culminated in the selection of 41 studies considered appropriate for the review. The search strategy is shown below in Table 1 below.

The inclusion criteria included studies that explicitly examined the mechanisms, clinical effects, comparisons, and safety profiles of slow-release treatments for glaucoma. The analyzed publications encompassed preclinical studies, clinical trials, and observational studies that examined various drug delivery platforms, including topical agents, ocular inserts, periocular devices, intracanalicular inserts, subconjunctival injections, supraciliary delivery systems, suprachoroidal delivery routes, and intracameral devices. Both innovative technologies and those recently sanctioned by the FDA were incorporated to furnish a comprehensive picture of the present landscape of slow-release treatments in glaucoma care. The methodological quality of each study was meticulously evaluated, focusing on study design, sample size, statistical analysis, follow-up duration, and reported outcomes.

Studies were excluded if they did not primarily address slow-release treatments for glaucoma, lacked relevance to the evaluation of the clinical impact and safety profiles of these therapies, or were not published in English. Furthermore, papers that could not be accessed due to the absence of complete texts were omitted. Articles that did not include original data were excluded to guarantee that the conclusions of this study were derived from primary research.

## 3. Results

This review identified various unique methods for slow-release medication delivery systems in glaucoma treatment. Hydrogel-based formulations and nanoparticulate systems among topical medications exhibited significant potential for improving bioavailability and facilitating prolonged drug release. Systems like SoliDrop and DuraSite ISV-215 have demonstrated successful lowering of intraocular pressure (IOP) with extended retention on the ocular surface. Nanoparticulate treatment, encompassing nanoparticles, liposomes, and PLGA nanocapsules, demonstrated potential for improving corneal penetration and prolonging drug delivery.

The Bimatoprost Ocular Ring Insert and TODDD™ exhibited promising outcomes regarding sustained intraocular pressure reduction, while certain limits concerning patient pain and device retention were observed. Periocular devices, such as punctal plugs and intracanalicular inserts, exhibited differing levels of effectiveness, with some showing extended efficacy and favorable retention rates, while others were linked to sporadic irritation and hyperemia.

Subconjunctival devices and injection-based therapies exhibited considerable promise for prolonged intraocular pressure reduction, especially in studies utilizing dorzolamide-loaded microparticle polymers, POLAT-001 nanoliposomes, and the IBI-60089 latanoprost injection system. In preliminary research phases, suprachoroidal and supraciliary delivery methods have shown encouraging outcomes for localized medication administration with diminished systemic adverse effects.

Intracameral devices like Durysta™ and iDose travoprost have demonstrated significant efficacy in clinical trials, achieving effective intraocular pressure reduction over prolonged durations with generally good safety profiles. The continuous delivery of bimatoprost and travoprost from these devices has been shown to facilitate long-term glaucoma care without requiring daily eye drop use.

This research indicates that slow-release medicines may substantially boost treatment adherence, minimize side effects, and improve clinical outcomes in glaucoma care. Nonetheless, the analysis underscores the necessity of performing additional extensive clinical trials to ascertain the long-term safety and usefulness of these novel technologies.

## 4. Discussion

### 4.1. Extraocular Drug Delivery Platforms

These refer to drug preparations utilized on the surface of the eye, which is the most common site for ocular therapeutics. They can be broadly classified as topical agents or ocular inserts.

#### 4.1.1. Surface Devices/Topical Agents

*i. Hydrogel* Hydrogel-based formulations stand as promising drug delivery systems for glaucoma treatment. They ensure prolonged drug release while minimizing systemic absorption and adverse effects. These systems can be fabricated using either physical or chemical methods of network formation. In the case of physical cross-linking, the polymer matrix is stabilized through non-covalent interactions, including ionic attractions, hydrogen bonds, or hydrophobic associations. Conversely, chemical cross-linking involves the formation of permanent covalent linkages within the polymer network. Their ability to encapsulate therapeutic molecules within a cross-linked polymeric matrix allows for controlled and sustained ocular drug delivery. Various types of hydrogels have been developed to improve retention time in ocular applications [4,5,6].

Nowadays, stimuli-responsive hydrogels—often referred to as “smart” hydrogels—have attracted growing attention due to their ability to mimic physiological feedback mechanisms. These materials undergo noticeable shifts in their physical properties in response to specific external factors such as temperature fluctuations or pH changes. For example, thermo-responsive hydrogels are typically formulated to be administered as a liquid that undergoes a phase transition to form a gel when exposed to specific temperature changes. In ophthalmic applications, these hydrogels are designed with gelling temperatures that align closely with the physiological temperature of the cornea.

A notable innovation in this field is the gel–microsphere (GMS) system developed by Fedorchak et al., known as SoliDrop (Otero Therapeutics, Pittsburgh, PA, USA) [7,8]. This noninvasive strategy merges thermo-responsive hydrogels with drug-encapsulating polymer microspheres, such as poly(lactic-co-glycolic acid) (PLGA), to form a depot that can be conveniently applied as a standard eye drop. Upon reaching a temperature of 33.5 °C, the hydrogel expands, sheds surplus water, and transitions into a semi-solid state, allowing it to stay in place on the eye surface for up to 28 days. The hydrogel/microsphere eye drop formulation enables extended release of brimonidine, maintaining therapeutic levels on the ocular surface without the need for repeated administration. The microspheres released an average of 2.1 ± 0.37 mg brimonidine tartrate per mg of microspheres per day, with an average volume diameter of 7.46 ± 2.86 μm. This release rate is therapeutically significant as it aligns with the daily dosing requirement of conventional brimonidine therapy, thereby validating the system’s efficacy for once-monthly dosing. In a study by Fedorchak et al., SoliDrop demonstrated a 30% IOP reduction in rabbits maintained for 28 days, matching the efficacy of twice-daily brimonidine drops [7].

Another advanced system is DuraSite ISV-215, which utilizes pentablock copolymers to deliver bimatoprost over prolonged periods [9]. The FDA-approved copolymers, composed of polyethylene glycol, poly(caprolactone), and polylactic acid/polyglycolic acid, form a mucoadhesive matrix that transforms into a gel upon contact with the ocular surface.

This gel-based platform has demonstrated significantly improved drug delivery, achieving a 2.2-fold increase in drug levels within the aqueous humor and a 6.1-fold enhancement in concentration within the iris and ciliary body of pigmented rabbits when compared to standard bimatoprost formulations. Depending on the administered dose, the release profile of the drug can be extended for up to six months. The system operates through gel expansion and the generation of hydrostatic pressure, which together support a continuous and controlled release of the active compound.

These hydrogel-based systems exemplify the potential of gels in glaucoma treatment. Their ability to provide sustained drug release, enhance bioavailability, and improve patient compliance underscores their significance as alternatives to conventional eye drops.

*ii. Nanoparticulate* Nanoparticulate systems for drug delivery, such as nanoparticles, nanoemulsions, nanosuspensions, liposomes, niosomes, and nanocrystals, have been widely researched for ocular applications. Their distinct characteristics, including mucoadhesive properties, improved corneal penetration, and the ability to provide prolonged drug release, make them highly effective for topical eye treatments [10]. These include nanoparticles, nanoemulsions, nanosuspensions, liposomes, and PLGA nanoparticles. These systems exhibit features like mucoadhesion, corneal penetration, and sustained release. Liposomes enable effective combination therapies and PLGA nanoparticles aid controlled drug release. Examples include brinzolamide-loaded PLGA capsules for 10-day IOP lowering effects and advanced lipid carriers for improved drug loading and permeability.

These systems are designed for both topical and intraocular drug delivery and are currently under investigation for their clinical efficacy in treating glaucoma. The topical application of nanoparticles, which exhibit mucoadhesiveness, high drug-loading capacity, and improved corneal permeability, has indeed shown promise for long-term glaucoma management. Composite formulations, such as nanoparticle-loaded hydrogels, further enhance corneal retention and provide sustained release of therapeutic agents during overextended periods [11].

One of the primary challenges with nanoparticulate drug delivery systems is the rapid initial release, or burst effect, which limits the duration of their therapeutic action. To overcome this limitation, various strategies have been investigated, including the design of lipid-based nanoparticles and polymeric formulations with strong drug-binding capabilities [12].

Among these, liposomes have gained particular attention as a promising vehicle for glaucoma therapies. These vesicles, formed by phospholipid bilayers, encapsulate drugs in a way that facilitates deeper penetration through the corneal tissue and ensures a more sustained release profile. This controlled release mechanism also enables the combination of multiple active agents, such as timolol maleate and brimonidine tartrate, leading to a more pronounced reduction in intraocular pressure (IOP) compared to conventional treatments. Furthermore, liposomal formulations offer protection to the ocular surface, reducing irritation, and contribute to better patient adherence, positioning them as an invaluable tool in managing glaucoma [13,14].

Another innovative approach involves the use of PLGA (poly-lactic-co-glycolic acid) nanoparticles, biodegradable and biocompatible polymers that undergo hydrolysis in the body, producing low-toxicity metabolites such as lactic acid. Glycolic acid, the second primary metabolite of PLGA degradation, is also biocompatible and naturally eliminated via the citric acid cycle, contributing to the overall safety of the formulation [15]. PLGA nanoparticles offer several advantages in ophthalmology, including protection against the external inactivation of loaded drugs and controlled drug release as the polymer degrades [15]. These nanoparticles exhibit high encapsulation efficiency (EE%) for both hydrophilic and hydrophobic drugs, reducing the frequency of drug administration in glaucoma treatment [16,17].

However, the hydrophobic nature and negative surface charges of PLGA nanocapsules can limit their mucoadhesive properties. This limitation can be addressed by modifying their surface area or applying mucoadhesive coatings, such as lipid-based or polymeric materials [18,19].

In a separate study, Shrivastava et al. developed nanostructured lipid carriers (NLCs) encapsulating brinzolamide and timolol maleate to enhance glaucoma therapy. The drugs were loaded into sesame oil, with the shell layer formed by Tween 80 and Precirol ATO 5 using a melt emulsification process. The optimized NLC formulations achieved drug loading (DL) and entrapment efficiency (EE) values of 0.71 ± 0.02% and 70.73 ± 0.64% for brinzolamide, respectively, and 0.360 ± 0.01% and 77.12 ± 0.64% for timolol maleate, respectively. In vitro release profiles showed initial burst release rates of 38 ± 3.10% and 34 ± 2.90% for brinzolamide and timolol maleate within 5 h, increasing to 70.08 ± 6.40% and 72.29 ± 5.90% after 24 h, respectively. Ex vivo corneal penetration studies confirmed significantly enhanced drug release and permeation from the NLCs compared to suspension formulations [20].

*iii. Contact lenses* Soft contact lenses, traditionally used for vision correction, have recently gained attention as a promising platform for ocular drug delivery. Composed of chemically cross-linked networks of hydrophilic polymers, these lenses are particularly suited for delivering hydrophilic drugs such as timolol and dorzolamide. However, the high hydration level of these materials results in rapid drug release, presenting a challenge for sustained release [21]. Their widespread patient acceptance and ease of use have positioned them as a viable alternative to conventional eye drops, particularly for glaucoma management [22].

The simplest method of drug loading, known as the soak-and-release technique, involves immersing contact lenses in a drug solution. This approach has been used to deliver anti-glaucoma medications such as pilocarpine, brimonidine, and timolol. While cost-effective and straightforward, this method is limited by a low drug-loading capacity and short duration of effect, typically lasting no more than 24 h. Moreover, the rapid initial release followed by a sharp decline in drug concentration renders it less suitable for chronic conditions like glaucoma, where sustained release is critical [23]. Researchers have developed advanced strategies to address these limitations. One such approach involves incorporating vitamin E (tocopherol) into contact lenses.

Researchers have explored innovative methods to overcome these challenges. One such method involves the incorporation of vitamin E (tocopherol) into contact lenses. Vitamin E serves as a lipophilic barrier, slowing down drug diffusion and extending the release period. Its antioxidant properties further protect the cornea from UV damage and prevent drug oxidation. Studies on beagle dogs, used as a model for glaucoma, indicate that contact lenses infused with vitamin E can extend the release of timolol and dorzolamide for up to two days, resulting in improved intraocular pressure (IOP) reduction compared to traditional eye drop formulations. When worn continuously for two days, followed by a replacement with a fresh set of drug-loaded lenses for an additional two days, these lenses sustain IOP reduction for up to four days, with effects lasting as long as eight days after removal [24].

Another innovative approach is the use of film-impregnated contact lenses. Ciolino et al. developed latanoprost-eluting contact lenses incorporating poly-(lactic-co-glycolic acid) (PLGA) film of varying thickness within methafilcon A lenses. These lenses demonstrated an initial burst release followed by sustained latanoprost release over one month. In vivo studies on glaucoma using monkeys showed that high-dose lenses (149 μg) reduced IOP by 6.0 ± 4.4 to 10.2 ± 2.5 mmHg, low-dose lenses (97 μg) reduced IOP by 4.0 ± 1.1 to 7.8 ± 3.8 mmHg, and latanoprost eye drops reduced IOP by 2.9 ± 1.0 to 6.6 ± 1.3 mmHg. The lenses provided sustained latanoprost release for one month, with aqueous humor drug concentrations comparable to those achieved with conventional drops [25].

Molecular imprinting has also been investigated as a method to improve drug delivery. This technique involves creating specific cavities in the lens material that exhibit a strong affinity for target drugs. For example, the study by Hiratani et al. demonstrated that contact lenses loaded with timolol, prepared using molecular imprinting, could sustain drug release for over 24 h in a saline solution [26]. Another promising strategy involves the use of supercritical fluids, which enhance the drug loading process and prolong the release duration for both hydrophilic and hydrophobic drugs [27].

Despite these advancements, challenges remain. Prolonged wear of drug-eluting contact lenses, especially overnight, can compromise oxygen permeability, leading to corneal edema [28]. Balancing effective drug release, IOP reduction, lens transparency, and oxygen permeability remains a significant hurdle. A summary of these delivery routes is listed below in Table 2.

#### 4.1.2. Ocular Inserts

In the 1970s, the first sustained-release device to ever be approved by the FDA for the treatment of glaucoma, Ocusert, consisted of a non-degradable ethylene-vinyl acetate microporous membrane containing pilocarpine as an active principle, surrounded by a ring of titanium dioxide. It was designed to be positioned under the lower fornix, where at a rate of either 20 or 40 µg/hour, it released the active principles with an elliptical effusion for one week [29]. It proved the possibility of having a reliable, continuously effective, and commercially available device, which also allowed accurate dosing in the elderly and children; however, it was eventually discontinued by the manufacturer 2 decades later, mainly because of the high costs and patient discomfort, including a bruising sensation and occasional dislocations.

Recently, a new ring device has been put forward for clinical use; the bimatoprost ocular ring insert consists of a non-biodegradable propylene ring surrounded by a silicone matrix containing bimatoprost, which is released with a progressively declining rate from 35 µg/day on the first day to 0–6 µg/day approaching the 180th day. Phase II trials on the bimatoprost ocular insert showed IOP reductions of 3.2 to 4.6 mmHg over 6 months, though with a 28% rate of insert dislocation [30].

The TODDD™ (Topical Ophthalmic Drug Delivery Device) is another eye insert device designed to sit in the upper eyelid area of the sclera and to release active principles over several months from a soft matrix depot. Preliminary trials reported a maximal 5.5 mmHg IOP drop, accounting for a 37% reduction compared to the baseline in beagles. Finally, a safety study in humans showed a 75% retention rate [31,32]. The advantages of these devices include continuous slow-release, reproducible kinetics, precise dosing of the active principle, minor diurnal fluctuations and prolonged activity in the target tissue, lower systemic absorption, and better patient compliance without requiring preservatives.

The disadvantages of these devices include foreign body sensations experienced by particularly sensitive patients, an initial burst release after installation before the controlled release, possible undesired migration, and rarely, difficulty placing or removing inserts.

### 4.2. Periocular Drug Delivery Platforms

Originally conceived to treat dry eye disease, lacrimal plug devices can be positioned in one or both puncta of each eye with the aid of a slit lamp to prevent the physiologic nasolacrimal drainage of tears. Thus, a higher volume of tears is maintained, which alleviates the symptoms of the disease. Punctual plugs, designed in different shapes, are temporary devices with a drug-diluting insert that allows a prolonged release. They are used to treat glaucoma.

*i. Intracanalicular Inserts* The OTX-TP consists of a rod-shaped hydrogel contained in an intracanalicular insert containing travoprost encapsulated inside polylactic acid microparticles for prolonged, gradual release for 90 days. Upon exposure to the tear film, this insert expands in the canaliculus to achieve a firm fixation; to make the insert visible, fluorescein is incorporated inside it for improved visualization under the slit lamp.

*Clinical safety and tolerability*

The results of a phase IIb study with 73 patients, comparing the device against a punctual plug eluting timolol (0.5%) twice a day and a placebo punctual non-eluting plug, showed promising results with a reduction in IOP of 4.5 to 5.7 mmHg compared to 6.4 to 7.6 in the control arm, with few side effects reported [33]. The results of the phase III trial in 554 subjects confirmed its efficacy and safety, leading to a 3.27–5.72 mmHg reduction, at least until week 12 of follow-up [34].

*ii. Punctal Plugs* The Evolute^®^ Punctual Plug Delivery System (PPDS) is an L-shaped device comprising a latanoprost-eluting polymer matrix enveloped in silicone. Initial phase 2 reports showed surprisingly high rates of device retention, with itching and a foreign body feeling as the most common side effects and the rare occurrence of hyperemia. Results over 12 weeks showed an encouraging 20% decrease from baseline in IOP [34,35].

The advantages of these devices include ease of plug insertion, prolonged permanence, little chance of failure, and the possibility of experimenting with new innovative approaches, such as nano- or microparticles that can be potentially inserted within the plug’s structure. The main limitation regards the system’s storage capability, which only allows the use of drugs active at a low dose, such as prostaglandins. Possible side effects include slight irritation or scratchiness in the tear duct and the rarer occurrence of inflammation, watery eyes, or allergic reactions.

*iii. Subconjunctival Injections/Devices* The subconjunctival route (including cul-de-sac or conjunctival fornix delivery systems) offers an appealing route for prolonged drug delivery, considering its proximity to tissues of the anterior segment implicated in the pathogenesis of ocular hypertension, such as cornea, limbus, and ciliary body.

The advantages of this route comprise the different potential delivery systems, including microspheres, gels, liposomes, and implants, with larger particles appearing more suitable for retention. There is also the possibility of having both pre-formed and in situ-forming implants for retention of particles over prolonged periods of time, potentially adding up to a few months. This approach has downsides, including the possibility of the dosage being externally visible to onlookers and causing hemorrhages on the eye surface while still being extremely unlikely to interfere with the patient’s visual axis [36,37,38]. Salama et al. developed a brinzolamide (BRZ)-loaded PLGA nanocapsule that demonstrated extended IOP-lowering effects for 10 days in vivo on rabbits’ eyes following a single subconjunctival injection [17].

The Eye-D VS-101, a latanoprost-based device, can be placed on the inferior bulbar conjunctiva with an outpatient procedure. Early trials showed a promising 24% reduction from baseline in IOP that was maintained over 12 weeks. It also achieved safety and efficacy endpoints, even though the exact drug dosage used was not yet specified by the manufacturer [39,40].

Injections of dorzolamide-loaded microparticle polymers can be performed in the subconjunctival space using a 27-gauge needle. Preliminary studies using Dutch rabbits on this ion-paired carbonic anhydrase inhibitor (dorzolamide) paired with a polylactic-co-glycolic acid polymer encapsulated inside a poly(ethylene glycol)-co-poly(sebacic acid) have shown promising results with an IOP reduction of 4.0 ± 1.5 mmHg compared to the control eye, maintained over 35 days [41].

The POLAT-001 was conceived as a 100 nm long nanoliposome containing latanoprost to be injected once in the subconjunctival space. POLAT-001 achieved over 20% IOP reduction at all follow-ups for 3 months in human studies [42].

The GB-6249-103 is a drug-encapsulating microparticle that contains new molecular entities (NME) based on different prodrugs of already approved IOP-lowering agents. A preclinical study using pigmented rabbits showed that a 20% IOP reduction was maintained from baseline for over two months, setting the stage for further evaluation [43].

Finally, the IBI-60089 device containing latanoprost allows for a subconjunctival liquid injection on a carrier platform, with the goal of achieving a six-month sustained release [44].

*iv. Supraciliary/Suprachoroidal* Hollow microneedles (or traditional needles) can be used for the delivery and prolonged release of drugs. The supraciliary route relies on penetration just beyond the sclera in the supraciliary space, the anterior-most region of the suprachoroidal space, allowing the needle to enter and for drug deposition to happen above the ciliary body, producing direct action on aqueous humor production. This route was exploited by Chiang et al. in a preclinical study by injecting poly(lactic acid) microspheres loaded with brimonidine supraciliary in New Zealand white rabbits. Results showed a 6 mmHg decrease in the study groups, which could be maintained for 14 and 33 days, depending on the administration of either the low- or high-dose formulation [45]. The advantages of this approach include the possibility of using less concentrated active principles (particularly compared to topical formulations) given their proximity to the target site of action.

For the suprachoroidal delivery route, the needle enters further away from the cornea and limbus, reaching closer to the choroidal region. This pathway has been particularly promising for drugs with physiochemical properties close to 1% hyaluronic acid (compound model), with data suggesting that it could be an incredibly efficient delivery route [46].

### 4.3. Intraocular Drug Delivery Platforms

*i. Intracameral* Durysta™ (Allergan, an AbbVie company, North Chicago, IL, USA) is the first intracameral delivery implant developed by Allergan Plc for the management of primary open-angle glaucoma (POAG) approved by the FDA in 2020 [47,48,49]. The biodegradable implant is designed for anterior chamber injection through a prefilled injector.

The NOVADUR drug delivery platform, previously approved for dexamethasone implant in 2009, allows intracameral implantation of 10 μg of bimatoprost via a 28-gauge disposable applicator [50,51].

Bimatoprost is incorporated into a biodegradable PLGA polymer matrix that undergoes hydrolysis, leaving no residue in the eye [52,53].

Compared with topical therapy (bimatoprost 0.03%), the implant provides an IOP-lowering effect that lasts for 4–6 months by producing a higher dependent concentration of the drug, specifically at the ciliary body receptors [54,55].

The APOLLO Phase I/II trial (2010–2016) evaluated the safety and efficacy of bimatoprost implants. It compared different doses of intracameral bimatoprost implants (6–20 μg) with topical therapy, finding that the implant provided a dose-dependent reduction in IOP. Adverse events were mostly mild and related to the implantation procedure. Moreover, typical side effects for prostaglandin drops, such as eyelash growth and iris hyperpigmentation, were not observed in any patients.

ARTEMIS 1 and 2 (two Phase III trials) tested 10 μg and 15 μg doses of the implant, comparing them to topical timolol. Results showed non-inferiority to timolol in IOP reduction. The implant’s effect remained consistent across repeated doses, but adverse events, including corneal decompensation, were more common with repeated treatments and higher doses [56,57]. Based on the efficacy and safety profiles reported in the ARTEMIS 1 and 2 studies, the implant received FDA approval [48,58].

Durysta is approved for single administration in the treatment of POAG and ocular hypertension (OHT). Repeated implantations have been associated with adverse effects on the cornea. Ocular inflammation, Fuchs’ dystrophy, and patients with a history of corneal transplantation are the main contraindications to the implant. Because of the risk of migration, the implant is also contraindicated in cases of absent or broken posterior lens capsules, including individuals with aphakia [48].

*ii. iDose* Glaukos (San Clemente, CA, USA) developed the iDose Travoprost [47], an intracameral biocompatible titanium implant for managing POAG. This implant includes a reservoir inserted into the anterior chamber and anchored within the trabecular meshwork through a small corneal incision.

The drug delivery system allows travoprost to be released into the anterior chamber for one year. However, the placement procedure is more invasive than DURYSTA™, as well as other devices such as ENV515 and OTX-TIC, and required gonioscopy during insertion [58,59]. When the drug reservoir is depleted, it must be removed and replaced.

A phase II clinical trial has shown that the implant reduced IOP by 33% compared to topical timolol treatment (30%) over 12 weeks of insertion [40]. A second Phase IIb study showed IOP reduction over 24 months, with no significant adverse events reported [59].

In a phase III clinical trial (GC-010 and GC-012), 93% of patients with iDose travoprost implants were controlled with the same or fewer medications at 12 months, while 81% no longer needed topical medications. Safety results were positive after 12 months of follow-up. Transient iritis, together with conjunctival hyperemia, were the most common adverse events. No significant corneal complications, such as endothelial cell loss were reported [60]. In February 2023, Glaukos submitted a device application for FDA approval.

Table 3 shows the essential characteristics of various slow-release drug delivery systems, offering a more lucid comparison than the descriptive analysis originally included in the manuscript. Each system demonstrates unique benefits and drawbacks that affect its clinical utilization. Microspheres and nanoparticles are highly efficient in targeted medication delivery, facilitating regulated distribution and extended effectiveness. Nonetheless, their stability poses a barrier, and there are apprehensions about potential toxicity, particularly at elevated doses. Liposomes have exhibited improved absorption, rendering them particularly advantageous in cancer treatment. Notwithstanding their benefits, they may provoke hypersensitive reactions, requiring vigilant oversight in clinical application.

Hydrogels have been developed as effective systems for localized and controlled drug delivery, especially in wound healing and regenerative medicine. However, their biocompatibility and degradation rates necessitate more modification to guarantee uniform treatment results. Implants, due to their sustained drug release functionality, markedly alleviate patient compliance issues by obviating the necessity for frequent doses. Nonetheless, their invasive characteristics and challenges in removal restrict their wider applicability. Transdermal patches offer a non-invasive method for drug delivery, facilitating continuous administration over prolonged durations. Notwithstanding their convenience, they encounter obstacles associated with medication permeability limitations, which may impact treatment efficacy.

## 5. Conclusions

Topical medication remains the first line of treatment for glaucoma; however, sustained ocular drug delivery via topical administration is difficult to achieve. Most drugs have poor penetration due to the multiple physiological barriers of the eye and are rapidly cleared when applied topically. Currently, daily topical administration for lowering IOP has many limitations, such as poor patient compliance and ocular allergy from repeated drug administration. Poor compliance leads to suboptimal control of IOP and disease progression with eventual blindness. The new drug delivery systems reported in this review provide a viable alternative by releasing medication to specific tissues gradually over an extended period, significantly reducing the frequency of dosing with minimal local toxicity and inconvenience. Sustained release platforms for the delivery of ocular hypotensive drugs (small molecules and biologics) may improve patient adherence and prevent optic nerve damage and vision loss. Furthermore, these new therapeutic options targeting drug delivery to specific ocular tissues reduce systemic exposure, thus minimizing side effects, above all in patients affected by systemic diseases or those taking multiple medications. Such innovations will only be widely adopted when efficacy and safety have been established using large-scale trials. It is expected that these approaches will improve the clinical management and prognosis of patients with glaucoma.

## Figures and Tables

**Table 1:** Search strings used for the literature search.

Keyword	Search String
Ocular	“ocular”[All Fields] OR “oculars”[All Fields]
Release	“patient discharge”[MeSH Terms] OR (“patient”[All Fields] AND “discharge”[All Fields]) OR “patient discharge”[All Fields] OR “release”[All Fields] OR “released”[All Fields] OR “releases”[All Fields] OR “releasing”[All Fields]
Therapies	“therapeutics”[MeSH Terms] OR “therapeutics”[All Fields] OR “therapies”[All Fields] OR “therapy”[Subheading] OR “therapy”[All Fields] OR “therapy”[All Fields] OR “therapy”[All Fields]

**Table 2:** Extracellular drug delivery routes.

Category	Details
**Topical Agents**	Hydrogel-based Gel-forming Drops: Promising for glaucoma treatment with prolonged drug release. Includes thermo-responsive hydrogels, gel–microsphere systems (SoliDrop), and DuraSite ISV-215. They offer sustained drug release, enhanced bioavailability, and improve patient compliance.
**Contact Lenses**	Chemically cross-linked hydrophilic polymers for delivering anti-glaucoma drugs like timolol. Advanced methods like vitamin E-loaded lenses extend release times and improve IOP reduction. These are a promising alternative to conventional eye drops but require periodic replacement for sustained effect.

**Table 3:** Summary of glaucoma-focused slow-release drug delivery platforms.

Delivery Platform	Route of Administration	Active Agent	Duration of Action	Development Phase	Clinical Outcomes
**Durysta™**	Intracameral (Intraocular)	Bimatoprost	4–6 months	FDA Approved	Non-inferior to timolol; well tolerated
**OTX-TP**	Intracanalicular (Periocular)	Travoprost	90 days	Phase III	3.27–5.72 mmHg IOP reduction; few side effects
**iDose**	Intracameral (Intraocular)	Travoprost	Up to 12 months	Phase III	93% medication-free at 12 months; safe profile
**Evolute PPDS**	Punctal Plug (Periocular)	Latanoprost	12 weeks	Phase II	20% IOP reduction; high retention, minimal irritation
**POLAT-001**	Subconjunctival (Periocular)	Latanoprost	3 months	Phase I	>20% IOP reduction; consistent across follow-ups
**IBI-60089**	Subconjunctival (Periocular)	Latanoprost	6 months	Preclinical	20% IOP reduction in rabbits for 60 days

## Data Availability

No new data were created or analyzed in this study. Data sharing is not applicable to this article.
